# 

**Published:** 1902-12-20

**Authors:** 


					the hospital. " The standard of highest purity."?Lancet. dec. 20th, 1902.
ir CADBURY'S ^ jg, CADBURY'S
COCOA V \? JP1I m J COCOA
ABSOLUTELY PURE. NO DRUGS OR ALKALIES USED.
scan Western Infirmary
No. 847. Vol. XXXIII. Lr&wspaperJ
Saturday, Dec. 20th, 1902.
L eeePp l92 j PRICB TWOPENCE.

				

